# Hypoxic tubular epithelial cells regulate the angiogenesis of HMEC-1 cells via mediation of Rab7/MMP-2 axis

**DOI:** 10.18632/aging.203648

**Published:** 2021-10-25

**Authors:** Yiqiong Yang, Jing Wang, Yu Zhang, Xiuxiu Hu, Li Li, Pingsheng Chen

**Affiliations:** 1 Department of Pathology and Pathophysiology, Medical School, Southeast University, Nanjing 210009, Jiangsu, China; 2 Institute of Andrology, The Affiliated Drum Tower Hospital, Nanjing University, Nanjing 210008, Jiangsu, China; 3 Institute of Nephrology, The Affiliated Zhongda Hospital, Southeast University, Nanjing 210009, Jiangsu, China

**Keywords:** Rab7, MMP-2

## Abstract

Renal hypoxia is associated with persisting peritubular capillary rarefaction in progression of chronic kidney disease (CKD), and this phenomenon mainly resulted from the dysregulated angiogenesis. Rab7 is known to be involved in renal hypoxia. However, the mechanism by which Rab7 regulates the renal hypoxia remains unclear. Protein expression was detected by western blot. Cell proliferation was detected by EdU staining. Cell migration was tested by transwell assay. Rab7 was upregulated in HK-2 cells under hypoxia conditions. Hypoxia significantly inhibited the viability and proliferation of human microvascular endothelial cells (HMEC-1 cells), while this phenomenon was obviously reversed by Rab7 silencing. Consistently, Hypoxia significantly decreased the migration and tube length of HMECs, which was partially reversed by knockdown of Rab7. Moreover, hypoxia-induced inhibition of MMP2 activity was significantly rescued by knockdown of Rab7. Moreover, ARP100 (MMP-2 inhibitor) significantly reversed the effect of Rab7 shRNA on cell viability, migration and angiogenesis. Furthermore, knockdown of Rab7 significantly alleviated the fibrosis in tissues of mice. Knockdown of Rab7 significantly alleviated the renal hypoxia in chronic kidney disease through regulation of MMP-2. Thus, our study might shed new light on exploring the new strategies against CKD.

## INTRODUCTION

Chronic kidney disease (CKD) is health problem with more than 70 million cases reported [1]. Although the prevalence of CKD varies in different countries and regions, the prevalence of CKD in developed countries has been increased to 11%. It is known that the deposition of immune complex, hypertension, hyperglycemia can lead to the progression of CKD, which results in cell damage and chronic inflammation in kidney with fibroblast activation and increase of extracellular matrix synthesis. Moreover, these phenomena can result in end-stage renal disease (ESRD). Hence, it is essential to explore the new methods for alleviating the fibrosis in CKD [2].

The reduction of peritubular capillary is a prominent pathological phenomenon in CKD progression; however, the underlying mechanism is still unclear. The renal tubular epithelial cells are the main cells of kidney and, they are extremely sensitive to hypoxia, proteinuria, toxins, metabolic disorders and senescence. It can release large numbers of active substances which are the primary causes of CKD, and these substances may perform different functions in status of peritubular capillaries [3]. It is confirmed that the decrease of peritubular capillaries may due to the main factors as follows: decreased blood flow through the peritubular capillaries decreased shear force on vascular wall and then weakened survival signals for blood flow-dependent endothelial and in the end caused endothelial cell apoptosis; the pericyte cells detached from the wall of the blood vessel because of local inflammation, followed with increased capillary permeability and instability, resulting in the capillary destruction; the microenvironment of peritubular capillaries changed in advanced disease, especially large amount of type I and type III collagen instead of less extracellular matrix and hindered exchange of information between cells and obstructed the status of VEGF [4–6]. Nevertheless, the effect of renal tubular epithelial cells injury on the formation of peritubular capillary has been largely unknown.

It has been previously reported that MMP-2 was a key factor of angiogenesis [7]. If hypoxic renal tubular epithelial cells were associated with these proteins, angiogenesis might be affected. Our previous study found that VEGF was upregulated in renal tubular epithelial cells under hypoxic condition, while the activity of MMP-2 was decreased [2, 8]. Therefore, the reduction of renal fibrosis tissue capillaries might result from the decrease of MMP-2 activity. Thus, we aimed to focus on the role of MMP-2 activity.

MMP-2 is an important member of matrix metalloproteinase family. It is majorly activated on the surface of cells. Many proteins participate in MMP-2 activity regulation, such as TIMP-2, RECK and MMP-14. TIMP-2 acts as a vital modulator in MMP-2 regulation, and TIMP-2 can bind with pro-MMP-2 to promote the activation of MMP-2 in low concentration; high concentration of TIMP-2 can inactivate MMP-2 [9]. On the other hand, RECK protein in cell membrane can inhibit the activity of MMP-2. MMP-2 has a wide range of functions after activation and can be degraded up to 27 extracellular substances. It can also play an anti-inflammatory role by antagonizing phospholipase A2 and monocyte chemotactic protein 3 [10].

Since the activation of MMP-2 is mainly carried out on the surface of plasma membrane, the change of membrane structure might affect the activity of this enzyme. Autophagy and endocytosis are main factors of cell membrane remodeling, and they can play important roles in regulation of transmembrane signal transduction. Hypoxia can induce the autophagy and endocytosis [2, 8], especially membrane receptor-related endocytosis [11]. Endocytosis and autophagy were closely correlated in hypoxia condition [2].

Rab7 is a crucial regulator of endosomal membrane transportation. In addition, Rab7 is proved to be related with autophagy vesicles, which is essential for the maintenance of physiological autophagy [12]. Rab7 can regulate the maturation of endosomes and autophagosomes, and it participates in the fusion of autophagy endocytosis and lysosomes. Thus, Rab7 is a key regulator in autophagy [13]. Nevertheless, the detailed mechanism by which Rab7 modulates the angiogenesis in CKD remains unclear. Based on the above background, we aimed to investigate the function of Rab7 in CKD. We hope this study would shed new light on finding the new methods for the treatment of CKD.

## RESULTS

### The expression of Rab7 was upregulated in HK-2 cells under hypoxia condition

**Figure 1:**
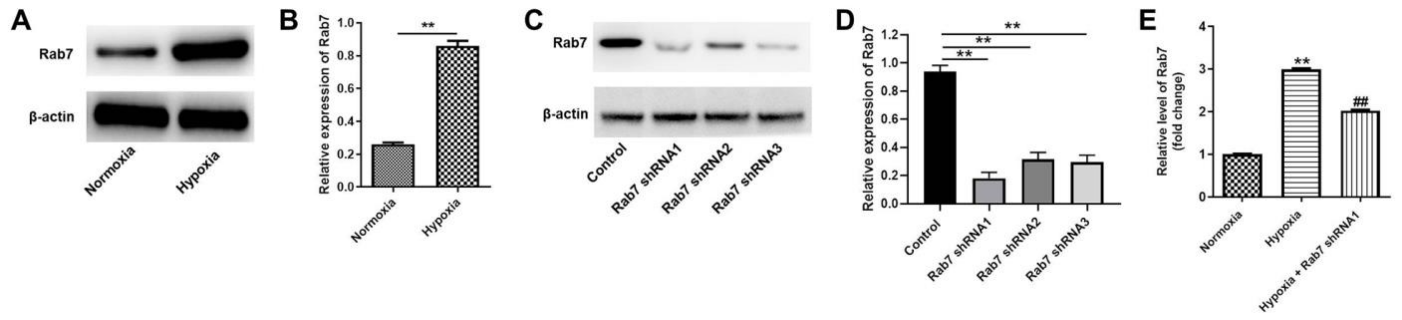
**Rab7 expression was upregulated in HK-2 cells under hypoxia condition.** HK-2 cells were maintained in normoxia or hypoxia condition. Then, (**A**, **B**) the protein expression of Rab7 in HK-2 cells was detected by western blot. β-actin was used for quantification. (**C**, **D**) HK-2 cells were transfected with Rab7 shRNA1, Rab7 shRNA2 or Rab7 shRNA3. The protein level of Rab7 in HK-2 cells was investigated by western blot. β-actin was used for quantification. (**E**) HK-2 cells in hypoxia condition were treated with Rab7 shRNA1. The level of Rab7 in HK-2 cells was tested by RT-qPCR. ^**^P<0.01 compared to normoxia or control. ^##^P<0.01 compared to Hypoxia.

To investigate the role of Rab7 in renal hypoxia in CKD, western blot was performed. As indicated in Figure 1A, 1B, the level of Rab7 was significantly upregulated in hypoxia-treated HK-2 cells than normoxia condition. Meanwhile, the expression of Rab7 in HK-2 cells was greatly downregulated by Rab7 silencing (Figure 1C, 1D). In addition, HK-2 cells were more sensitive to Rab7 shRNA1, compared with Rab7 shRNA2 or shRNA3 (Figure 1C, 1D). Thus, Rab7 shRNA1 was selected in further analysis. Furthermore, hypoxia-induced upregulation of Rab7 was significantly reversed by Rab7 shRNA1 (Figure 1E). Taken together, Rab7 was upregulated in HK-2 cells under hypoxia condition.

### Knockdown of Rab7 reversed hypoxia-induced inhibition of HMEC-1 cell proliferation

**Figure 2:**
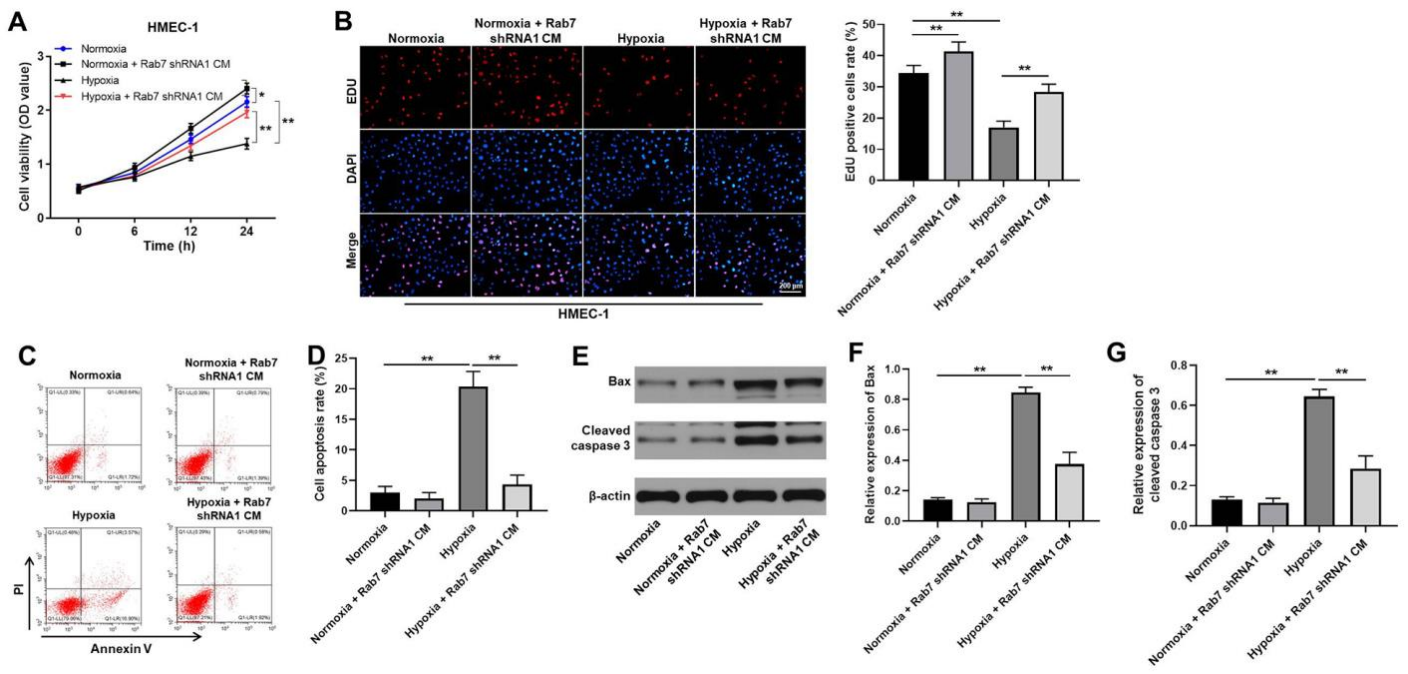
**Knockdown of Rab7 reversed hypoxia-induced inhibition of HMEC-1 cell proliferation.** In order to detect the function of Rab7 in hypoxia-treated HK-2 cells, cells were cultured in hypoxia and normoxia, respectively. Then, the cell supernatant was collected and cultured in conditioned medium (CM). After that, HMEC-1 cells were maintained in CM. (**A**) CCK-8 assay was performed to detect the viability of HMEC-1 cells. (**B**) The proliferation of HMEC-1 cells was tested by EdU staining. (**C**, **D**) The apoptosis of HMEC-1 cells was tested by flow cytometry. (**E**) The protein levels of Bax and cleaved caspase 3 in HMEC-1 cells were detected by western blot. (**F**, **G**) β-actin was used for quantification. ^*^P<0.05, ^**^P<0.01.

In order to detect the function of Rab7 in hypoxia-treated HK-2 cells, cells were cultured in hypoxia and normoxia, respectively. Then, the cell supernatant was collected and cultured in conditioned medium (CM). After that, HMEC-1 cells were maintained in CM. As demonstrated in Figure 2A, hypoxia notably inhibited the viability of HMEC-1 cells, while Rab7 shRNA1 obviously reversed this phenomenon. Consistently, the proliferation of HMEC-1 cells was significantly inhibited by hypoxia, while the anti-proliferative effect of hypoxia was rescued by knockdown of Rab7 (Figure 2B). Meanwhile, Rab7 knockdown could also increase the viability and proliferation of HMEC-1 cells under nomoxia condition (Figure 2A, 2B). Taken together, knockdown of Rab7 reversed hypoxia-induced inhibition of HMEC proliferation.

### Rab7 silencing reversed hypoxia-induced decrease of cell migration and angiogenesis

**Figure 3:**
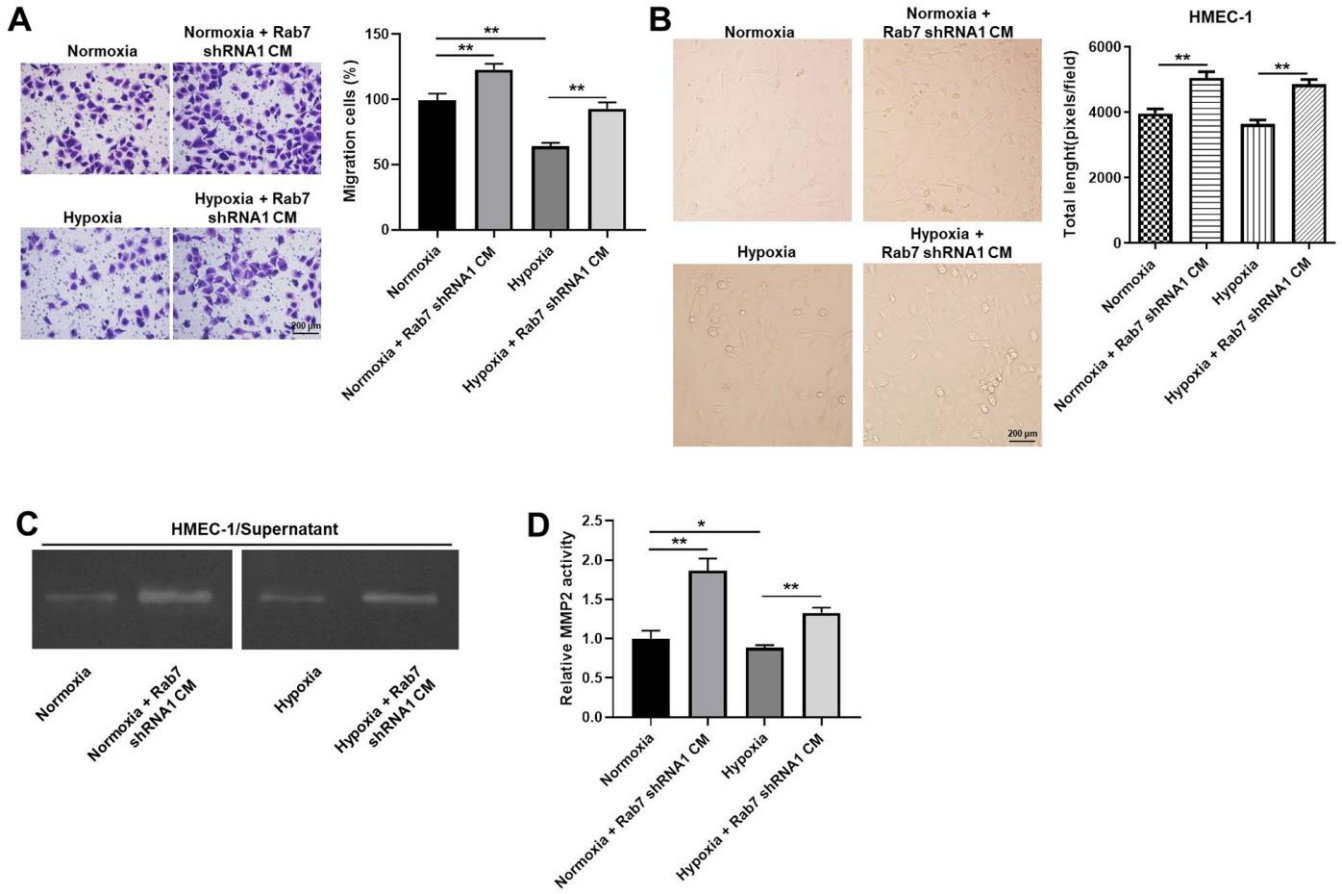
**Rab7 silencing reversed hypoxia-induced decrease of cell migration and angiogenesis.** (**A**) The migration of HMEC-1 cells was tested by transwell assay. (**B**) The angiogenesis of HMEC-1 cells was investigating by calculating the total length. (**C**, **D**) The MMP-2 activity in HMEC-1 cells was tested by Gelatin Zymography. ^*^P<0.05, ^**^P<0.01.

For the purpose of detecting the cell migration, transwell assay was used. The data indicated that the migration of HMEC-1 cells was significantly decreased by hypoxia, which was markedly rescued by downregulation of Rab7 (Figure 3A). Consistently, hypoxia-induced decrease of angiogenesis was partially reversed in the presence of Rab7 silencing (Figure 3B). Moreover, hypoxia obviously inhibited the activity of MMP-2 in HMEC-1 cells, while this phenomenon was markedly restored by Rab7 shRNA1 (Figure 3C, 3D). Thus, these data revealed that Rab7 silencing reversed hypoxia-induced decrease of cell migration and angiogenesis.

### Rab7 silencing reversed hypoxia-induced decrease of MMP-2 activity via regulation of RECK, MMP-14 and Caveolin-1

**Figure 4:**
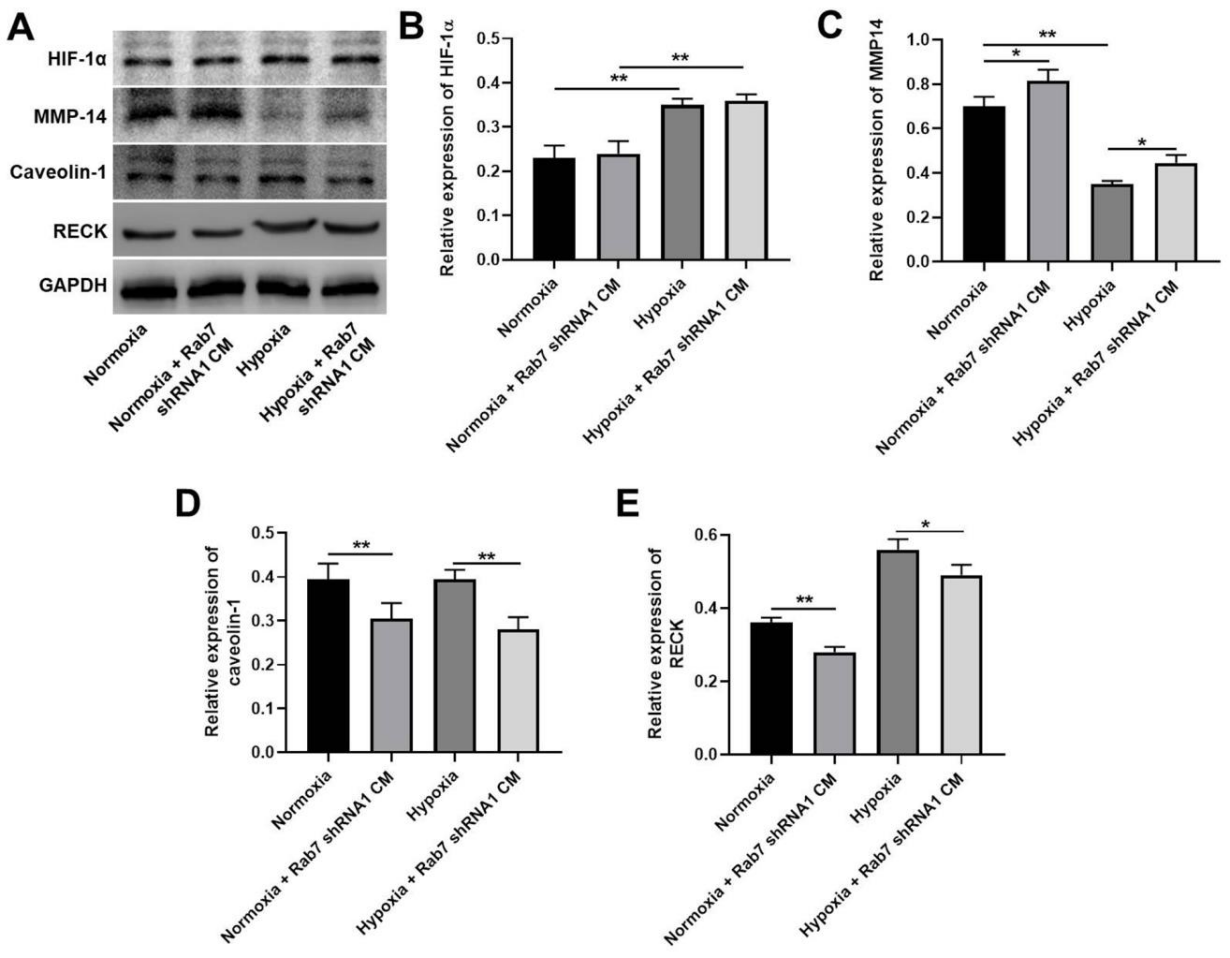
**Rab7 silencing reversed hypoxia-induced decrease of MMP-2 activity via regulation of RECK, MMP-14 and Caveolin-1.** (**A**) The protein levels of HIF-1α, MMP-14, Caveolin-1 and RECK in HMEC-1 cells were detected by western blot. (**B**–**E**) β-actin was used for quantification. ^*^P<0.05, ^**^P<0.01.

To investigate the mechanism by which Rab7 reverses hypoxia-induced inhibition of HK-2 cell proliferation, western blot was used. As shown in Figure 4A, 4B, hypoxia significantly upregulated the expression of HIF-1α in HK-2 cells, In contrast, hypoxia-induced decrease of MMP-14 was significantly rescued in the presence of Rab7 knockdown (Figure 4A, 4C). Meanwhile, Rab7 knockdown obviously inhibited the levels of Caveolin-1 and RECK in normoxia/hypoxia-treated HK-2 cells (Figure 4A, 4D, 4E). In summary, Rab7 silencing reversed hypoxia-induced decrease of MMP-2 activity via regulation of RECK, MMP-14 and Caveolin-1.

### Rab7 shRNA1 reversed hypoxia-induced decrease of migration and angiogenesis in HMEC-1 cells via upregulation of MMP-2 activity

**Figure 5:**
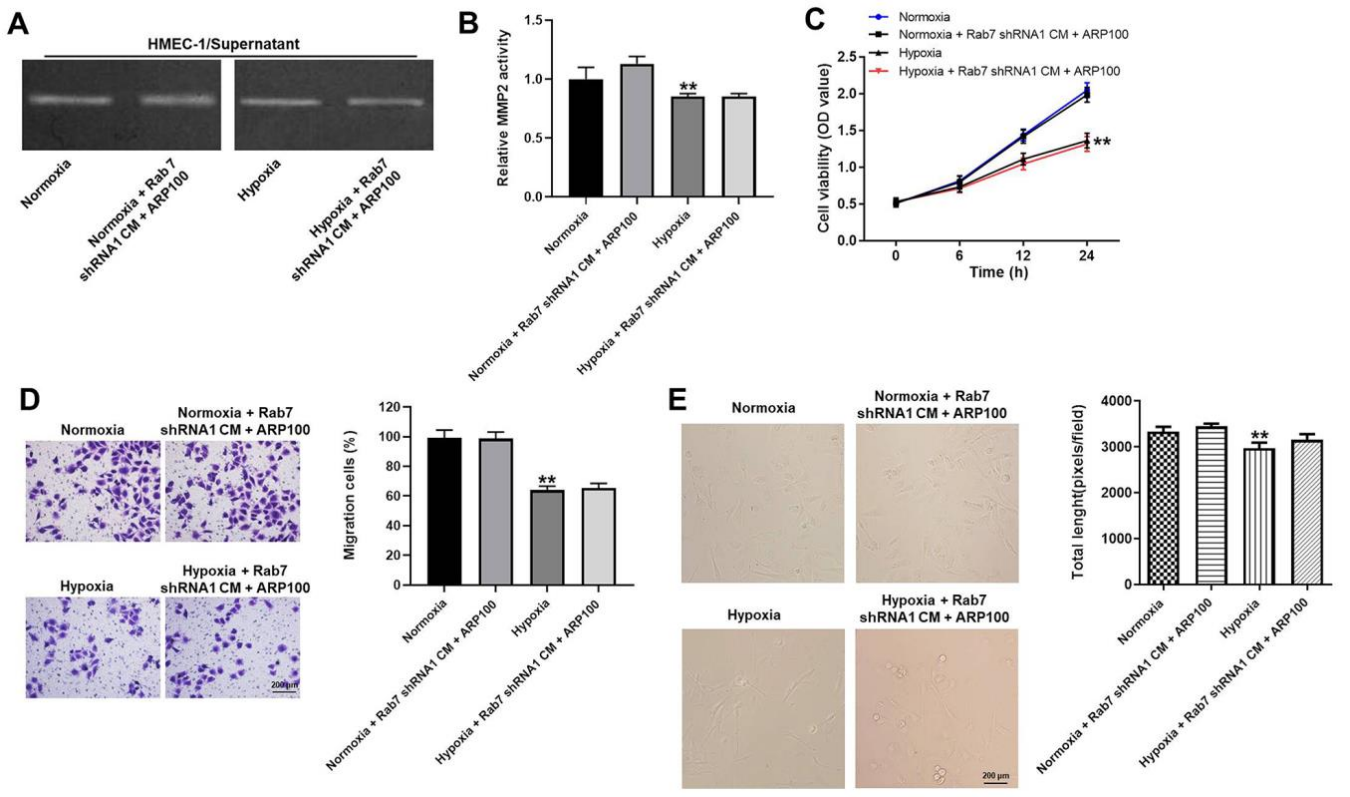
**Rab7 shRNA1 reversed hypoxia-induced decrease of migration and angiogenesis in HMEC-1 cells via upregulation of MMP-2 activity.** (**A**, **B**) The MMP-2 activity in HMEC-1 cells was tested by Gelatin Zymography. (**C**) The viability of HMEC-1 cells was tested by CCK-8 assay. (**D**) The migration of HMEC-1 cells was tested by transwell assay. (**E**) The angiogenesis of HMEC-1 cells was investigating by calculating the total length. ^**^P<0.01 compared to normoxia.

In order to investigate the correlation between Rab7 and MMP-2 in hypoxia-treated HMEC-1 cells, rescue experiments were performed. As revealed in Figure 5A, 5B, the activity of MMP-2 in normoxia/hypoxia-treated HMEC-1 cells was limitedly affected in the presence of Rab7 shRNA1 and ARP100. Consistently, Rab7 shRNA1 in combination with ARP100 had very limited effect on cell viability, migration and angiogenesis of normoxia/hypoxia-treated HMEC-1 cells (Figure 5C–5E). To sum up, Rab7 shRNA1 reversed hypoxia-induced decrease of migration and angiogenesis in HMEC-1 cells via upregulation of MMP-2 activity.

### Knockdown of Rab7 significantly alleviated the symptom of CKD *in vivo*

**Figure 6:**
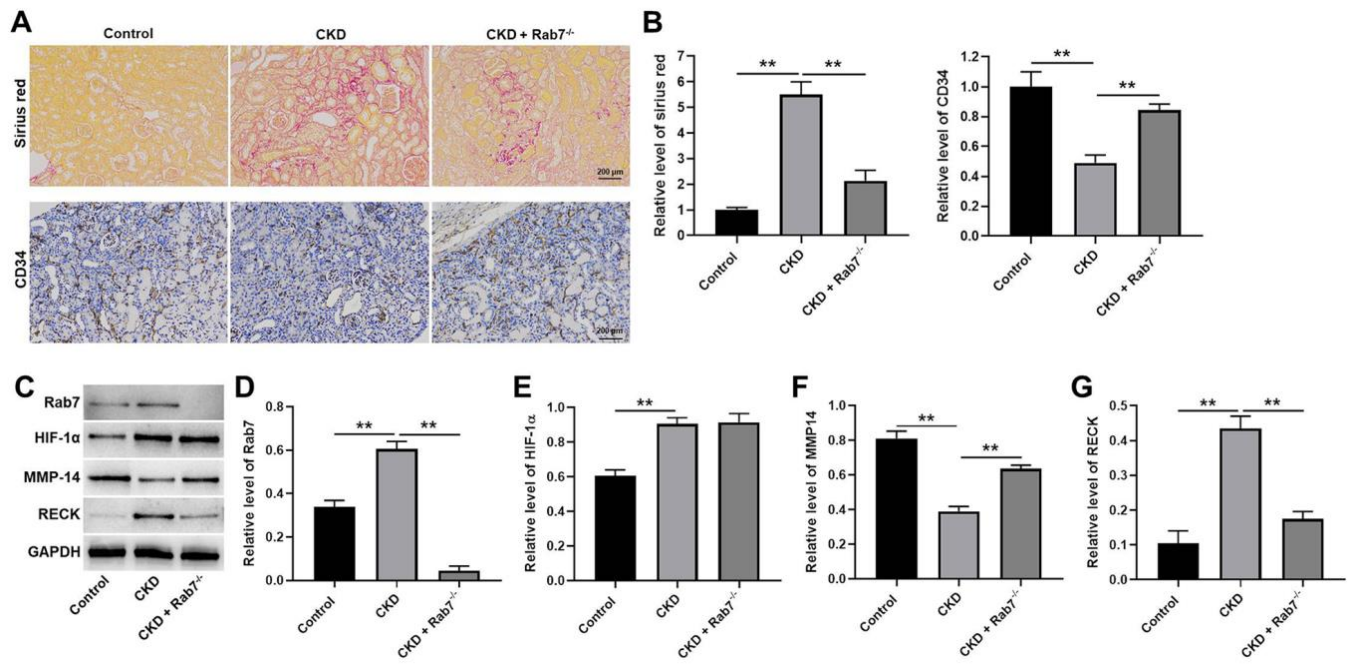
**Knockdown of Rab7 significantly alleviated the symptom of CKD *in vivo*.** (**A**, **B**) Sirius red staining was used to detect the fibrosis in kidney tissues of mice. The level of CD34 in kidney tissues of mice was detected by IHC staining. (**C**) The protein levels of HIF-1α, Rab7, MMP-14 and RECK in kidney tissues of mice were detected by western blot. (**D**–**G**) β-actin was used for quantification. ^**^P<0.01.

Finally, *in vivo* model of CKD was established to investigate the function of Rab7 in CKD *in vivo*. As revealed in Figure 6A, 6B, the relative level of Sirius red in kidney tissues of mice was significantly upregulated by CKD, while Rab7 knockdown reversed this phenomenon. In contrast, CKD-induced inhibition of CD34 level was partially rescued in the presence of Rab7 silencing (Figure 6A, 6B). Moreover, CKD significantly increased the levels of Rab7, RECK and inhibited the expression of MMP-14 in kidney tissues of mice, while Rab7 knockdown reversed these phenomena (Figure 6C–6F). However, Rab7 silencing did not affect CKD-induced upregulation of HIF-1α (Figure 6C, 6G). In summary, knockdown of Rab7 significantly alleviated the symptom of CKD *in vivo*.

## DISCUSSION

Peritubular capillary rarefaction is an important pathological feature of CKD, and it can lead to the occurrence of renal hypoxia [14]. Meanwhile, renal hypoxia can promote the development of CKD [14]. Therefore, it is important to promote the formation of peritubular capillaries and increase the supply of tissue oxygen for alleviating the progression of CKD [15]. Hypoxia is a prominent pathophysiological feature of renal fibrosis. Our previous data confirmed that enzyme activity of MMP-2 was decreased in hypoxic cultured renal tubular epithelial cell supernatants and renal fibrotic tissues [2, 8]; however, the mechanism by which enzyme activity was decreased under hypoxia condition needs to be explored. In this research, we found hypoxic tubular epithelial cells regulating regulates the angiogenesis of HMEC-1 cells by Rab7/MMP-2 axis. Thus, our study firstly explored the correlation between hypoxic tubular epithelial cells and HMEC-1 cells in CKD, which supplemented the function of Rab7.

As a shared molecule of autophagy and endocytosis in renal tubular epithelial cells, Rab7 can control the transport of late endocytosis to lysosomes [16, 17]. Rab7 plays an important role in pinocytosis, phagocytosis and exocytosis. Meanwhile, Rab7 is the basis of lysosomal biosynthesis, localization and function, and it also participates in many physiological activities such as apoptosis, neurotrophic factor transport and signal transduction [18]. Rab7 is a hinge molecule linking various functions inside and outside of the cell [19]. On the other hand, MMP-2 is mainly released from cells in form of proenzyme and the activation on the surface of cells [20, 21]. In addition, autophagy is the main factor of cell membrane remodeling, and it is known to play a key role in the regulation of transmembrane signal transduction and metabolic activities [22–24]. Our recent data indicated that endocytosis in hypoxic renal tubular epithelial cells could affect the activity of MMP-2 [25]. In addition, the rescue experiments indicated that ARP100 reversed the effect of Rab7 shRNA1 on cell proliferation and migration. Thus, our data further indicated the role of Rab7 in MMP2 activity. To sum up, Rab7 could act as a key regulator in CKD.

This study found the cultured supernatant of renal tubular epithelial cells with low expression of Rab7 could promote the proliferation of endothelial cells. Thereby, our study firstly explored the crosstalk between renal tubular epithelial cells and endothelial cells. Zou et al. found that the mesenchymal stromal cell-derived extracellular vesicles alleviated renal ischemic reperfusion injury [26]; Chade et al. indicated that polymer-stabilized vascular endothelial growth factor could promote renal angiogenesis [27]. Moreover, it was reported that protein kinase LKB1 promoted Rab7-mediated NRP-1 degradation and inhibited the angiogenesis in tumor cells [28]. In the recent work, we found that endothelial cells co-cultured with renal carcinoma cells significantly reduced RECK expression under chemical hypoxia [29]. Based on the above data, angiogenesis plays a crucial role in early and late stage of kidney diseases. Meanwhile, Wang CG et al. found that Asperosaponin VI could promote angiogenesis in rats via upregulation of HIF-1α/VEGF signaling [30]. Our data were similar to this previous study. VEGF was confirmed to be a crucial mediator in angiogenesis [31, 32]. Thereby, the similar function between VEGF and Rab7 might result in the similarity between our research and Wang CG et al.

Indeed, there are some shortcomings in this work as follows: 1) more mechanisms by which hypoxic tubular epithelial cells regulate the angiogenesis of HMEC-1 cells need to be explored; 2) the downstream target of Rab7 needs to be further explored; 3) the EMT markers should be further analyzed. Therefore, more investigations are needed in coming future.

In conclusion, hypoxic tubular epithelial cells regulate the angiogenesis of HMEC-1 cells by mediation of Rab7/MMP-2 axis. Thus, our research might shed a new light on exploring the new strategies for the treatment of CKD.

## MATERIALS AND METHODS

### Mice and reagents

Animal Care Committee of Southeast University (No. 2019101201) approved this study. Rab7 knockout mice were constructed by using CRISPR/Cas9 technology (SaiyeBiotechnology Co, LTD.). Wild-type mice (C57BL/6, 18-22 g) were bought from Qinglongshan Company (Nanjing, China). Mice were kept in a condition of SPF. Cyclosporine A (CsA, Chem Best, Shanghai, China) was diluted in sunflower oil (10 mg/ml, Arawana, Shanghai, China). Furosemide (10 mg/ml, ZhaoHui) was diluted in distilled water.

### Experimental design

The mice were classified into Control, CKD and CKD + Rab7^-/-^ group. In order to induce chronic renal fibrosis, mice in were treated with 50 mg/kg furosemide 2 days before the experiment and then administrated with 25mg/kg CsA and 50mg/kg furosemide daily for 28 days (except control group). Control mice were administrated with saline for four weeks.

Finally, mice were sacrificed with the anaesthetization of pentobarbital (Xiya, Linyi, China). Kidney tissues of mice were obtained for the investigation of histology and western blot. The experiments were done in line with NIH guide.

### Cell culture and treatments

HK-2 and 293T cell lines (human renal proximal tubular epithelial cell line) were obtained from the Chinese Academy of Sciences (Shanghai, China). HMEC-1 cells were obtained from Procell (Wuhan, China). HK-2 and 293T cells were maintained in medium (DMEM/F12, Gibco) containing FBS (10%, Gibco), streptomycin (100 μg/ml) and penicillin (100 U/ml, Gibco). In addition, the supernatants of HK-2 cells were collected and taken for conditioned medium (CM). HMEC-1 cells were cultured in CM. All cells were maintained in a condition of 37° C and 5% CO_2_.

### Cell transfection

Rab7 shRNA1 (Genepharma), shRNA2 (Genepharma) or shRNA3 (Genepharma) was packaged. After that, lentiviral vector DNAs were transfected into 293T cells including NC and lenti-Rab7 shRNAs. The supernatant was collected after cell transfection. Then, supernatants of three Rab7 shRNAs and negative control were filtered into particles. HK-2 cells were infected with lentiviral particles. Puromycin (2.5 μg/mL, Sigma Aldrich) was used to select the stable cells after incubation. Western blot was used to assess the transfection efficiency.

### Reverse transcription quantitative polymerase chain reaction (RT-qPCR)

TRIzol (Invitrogen) was used to isolate RNA from cells. PrimeScript RT Kit (Invitrogen) was applied to synthesize First-strand cDNA. SYBR Green methods were used in RT-qPCR detection with an ABI7500 system. The experimental condition for RT-qPCR was as follows: 95° C for 2 min, 40 cycles at 95° C for 30 sec, 58° C for 30 sec and 72° C for 1 min. The primers originated from GenePharma. 2^-ΔΔCT^ method was used to quantify the data. β-actin was used for quantification. The primers were obtained from GenePharma (Shanghai, China). Rab7: forward, 5’-TCTGGAGTCGGGAAGACATC-3’ and reverse 5’-CTGTCCTGCTGTGTCCCATA-3’. β-actin: forward, 5’-TCACCCACACTGTGCCCATCTACGA-3’ and reverse 5‘-CAGCGGAACCGCTCATTGCCAATGG-3’.

### *In vitro* angiogenesis assay

HMEC-1 cells (2×10^5^/ml) were cultured overnight. Subsequently, cells were incubated under hypoxic or normoxic condition for 2 h. Cell suspension (100 μL) was added to each well. Then, the tubule networks (capillary-like) were analyzed by investigating total length of the cells per field. The data was quantified by using Image J Software.

### Western blot

RIPA was used to extract total protein from tissues or cells. BCA kit (Beyotime, China) was used to quantify the proteins. SDS-PAGE (10%) was used to separate the proteins, and proteins were then transferred into polyvinylidene fluoride (PVDF) membranes. Primary antibodies were used to incubate the membranes overnight after blocked with 3% skim milk for 1 h. After that, secondary antibody (anti-rabbit, Abcam; ab7090, 1:5000) was used to incubate the membranes for 1 h. ECL (Thermo Fisher Scientific) was used to measure the protein bands. The antibodies were as follows: anti-Rab7 (Proteintech; 55469-1-AP, 1:1000), anti-HIF-1α (Proteintech; 20960-1-AP, 1:1000), anti-Caveolin-1 (Proteintech; 16447-1-AP, 1:1000), anti-Bax (Abcam; ab32503, 1:1000), anti-cleaved caspase 3 (Abcam; ab32042, 1:1000), anti-MMP-14 (Bioworld; BS7040, 1:1000), anti-RECK (CST; #3433S, 1:1000) and anti-β-actin (Proteintech; 20536-1-AP, 1:1000). β-actin was used as an internal control.

### CCK-8 assay

HK-2 cells, HK-2-Rab7^-^ cells were cultured under normal or hypoxic conditions, and the supernatants were collected and served as conditioned media. A hypoxia incubator (Thermo) was used to obtain a condition of hypoxic (5% CO_2_, 94% N_2_ and 1% O_2_). HMEC-1 cells were cultured in condition medium. According to the types of conditioned media (CM), the cells (4 × 10^3^ cells/well) were divided into 6 groups (Normoxia, Hypoxia, Normoxia + Rab7 CM shRNA1, Hypoxia + Rab7 shRNA1 CM, Normoxia + Rab7 shRNA1 CM +ARP100 and Hypoxia + Rab7 shRNA1 CM +ARP100. After that, cells were exposed to CCK-8 (Beyotime, China) and incubated for another 2 h, OD values were assessed at 450nm by a micro plate reader.

### Immunofluorescence

HMEC-1 cells were cultured in condition medium for 24h. After that, the cells were fixed with 4% par formaldehyde for 1 h, and then Triton X-100 (0.5%) was used to permeabilize the cells for 10 min. Nonspecific binding was blocked with sheep serum (10%, BOSTER). Primary antibodies against EdU (1:200) (Proteintech, China) was applied to incubate the cells overnight, and then the appropriate secondary antibody (1:1000, Thermo Fisher Scientific) was used to incubate the cells for 1 h. DAPI was used to stain the nuclei for 5 min. The result was observed by a microscopy (Olympus, Japan).

### Transwell assay

Cells (2.5×10^3^) were plated into the upper chamber in medium containing 1% FBS. The lower chamber was supplemented with the medium containing 20% FBS. After incubation, the transwell chamber was rinsed, fixed. After that, 0.1% crystal violet was used to stain the chamber for 30 minutes. The migrated cells were observed under a microscope.

### Gelatin zymography

After being cultured in the condition media for 24h, the endothelial cells were further cultured in free serum DMEM/F12 medium for 12h, and then the supernatants were collected. The activity of MMP-2 in the condition media and the supernatants of the endothelial cells were assessed by gelatin zymography (Applygen, China) [33]. The supernatants were collected after centrifugation and the content of protein in cell supernatants was detected by a BCA kit (Beyotime, China). The equal protein amount of supernatants (40μg) were separated by 8% poly acryl amide gel containing 0.1% bovine gelatin (Sigma, USA). After electrophoresis, the gel had been transferred to Triton X-100 solution (2.5%, 5 h) and then had been incubated in Tris-HCl buffer (pH 7.4) overnight. Coomassie Brilliant Blue R-250 (0.1%) was used to treat the gel. and white bands were visualized After the gel was destained in 30% methanol and 10% acetic acid, white bands were visualized. Finally, the data were analyzed by Image J Software.

### Cell apoptosis detection

Cells were trypsinized, washed and re-suspended in Annexin V Binding Buffer. Then, cells were stained with FITC (5 μl, BD) and 5 μl propidium (PI, BD) in the dark for 15 min. Flow cytometer (BD) was used to analyze the cells.

### Sirius red staining

After euthanasia, the kidney tissues were quickly removed, and kidney portion of samples were removed and frozen. Par formaldehyde (4%) was used to fix the remaining kidney tissues, and then tissues were embedded and sectioned. Sirius red staining was used to evaluate renal fibrosis in different groups.

### Immunohistochemistry stain

Tissues were fixed in 4% paraformaldehyde overnight, paraffin-embedded, and cut into sections (5-μm-thick). After sections were deparaffinized and rehydrated, they were heated for antigen retrieval. Next, sections were washed for 5 min, and 3% H_2_O_2_ was used to incubate the samples for 25 min. Then, samples were blocked and incubated for 30 min after washed for 5 min. Afterwards, primary antibodies (anti-CD34, 1:400; Proteintech, Rosemont, IL, USA) were applied to stain the samples overnight. Samples were then incubated with secondary antibody (HRP-labeled) for 30 min. Color was developed by DAB. The antibodies were bought from Abcam. The tissues were observed under a fluorescence microscope.

### Statistical analysis

Three independent experiments were done in each group, and mean ± standard deviation (SD) was applied to represent the data. Student’s t-test (only 2 groups) or one-way analysis of variance (ANOVA) followed by Tukey’s test (more than 2 groups, Graphpad Prism7) was applied to analyze the differences between different groups. P<0.05 suggested a great change.

### Ethical statement

The protocols for animal care and use of laboratory animals were in accordance with Medical School of Southeast University (No. 2019101201). All *in vivo* experiments were performed in accordance with National Institutes of Health guide for the care and use of laboratory animals.
